# Intracardiac ectopic thyroid mass: A case report

**DOI:** 10.1016/j.xjtc.2024.12.009

**Published:** 2025-01-10

**Authors:** Yuefeng Cao, Tingting Song, Yansong Zuo, Ying Zhao, Qiang Wang, Jun Yan

**Affiliations:** Pediatric Cardiac Center, Beijing Anzhen Hospital, Capital Medical University, Beijing, China

**Figure:**
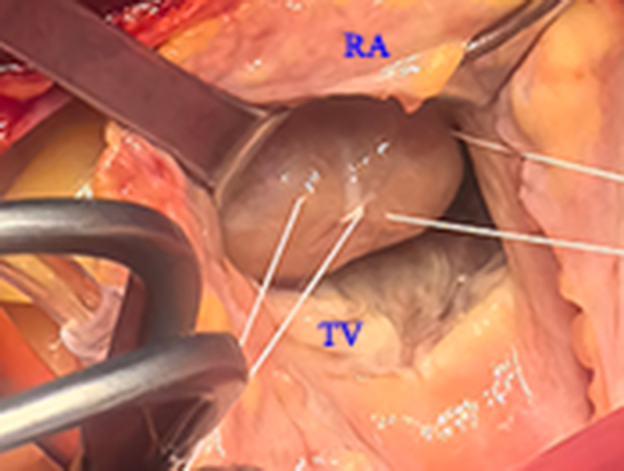
Intraoperative view showing a mass in the right ventricular outflow tract.

**Central Message**

We present a rare case of successful excision of intracardiac ectopic thyroid tissue obstructing the right ventricular outflow tract.

Ectopic thyroid tissue is a rare disease resulting from developmental defects of thyroid gland embryogenesis.^1^ It can be found in different locations in the body.^2^ Ectopic thyroid in the heart is a much rarer condition. We present a rare case of successful excision of intracardiac ectopic thyroid tissue obstructing the right ventricular outflow tract (RVOT).

## Case Report

**Figure 1:**
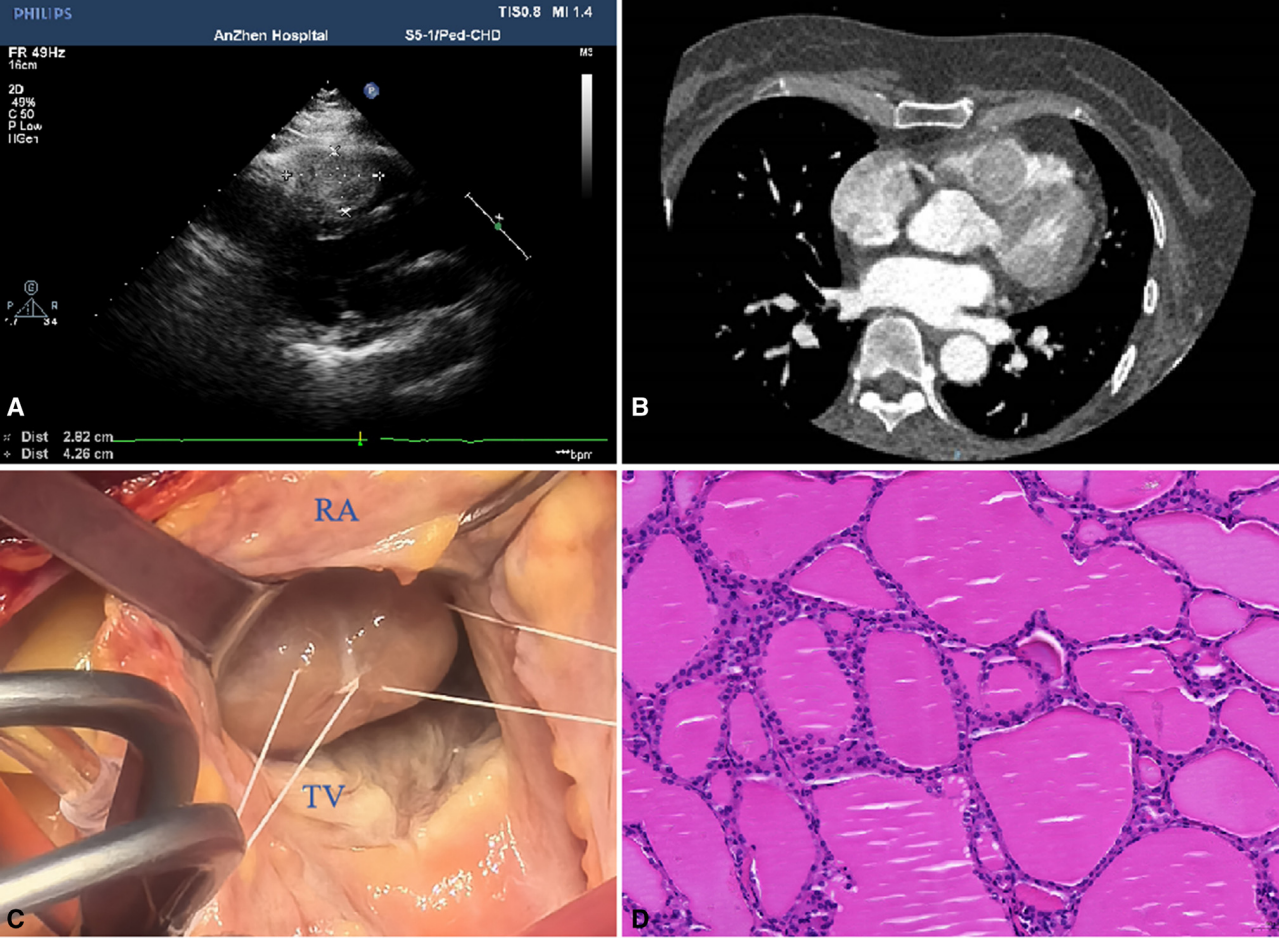
A, Transthoracic echocardiography demonstrating a right ventricular outflow tract (RVOT) mass arising from the septum. B, Initial computed tomography scan of the chest showing a mass in the RVOT. C, Intraoperative view showing a mass in the RVOT through the tricuspid valve. D, Photomicrograph of the nodule under higher magnification (×20) showing multiple thyroid follicles. *RA*, Right atrium; *TV*, tricuspid valve.

A 54-year-old woman reported a 2-year history of chest tightness, shortness of breath, and fatigue related to exercise. She denied weight loss during the past 2 years. She has a 3-year history of high blood pressure and takes regular medication for it. Echocardiography showed a large oval mass attached to the anterior wall of the right ventricle and the interventricular septum (Figure 1, *A*) resulting in severe obstruction of the RVOT. The mass had complete and clear borders and was slightly active with the cardiac cycle; there were no blood flow signals in the mass. Computed tomography (CT) was performed to further confirm the location of the mass and to exclude metastatic malignancy in the chest and abdomen (Figure 1, *B*). CT images showed a roundish, inhomogeneous-density mass in the right ventricle with a size of 2.9 × 2.67 cm.

The patient underwent surgery to resect the mass. At operation, standard cardiopulmonary bypass was performed. Cardiac arrest was achieved with crossclamp, antegrade blood cardioplegia, and mild hypothermia at 32 °C. During intraoperative exploration, a 4.3 × 2.8 cm mass was found that attached to the tricuspid papillary muscle and was adhered to the anterior wall of the right ventricle (Figure 1, *C*). The mass was removed intact through right atrial incision. After surgery, intraoperative transesophageal echocardiography showed that there was no shunt from the septum and RVOT obstruction. Histology revealed thyroid follicles of varying sizes filled with colloid (Figure 1, *D*).

## Comment

Ectopic thyroid is defined as the presence of thyroid tissue in a location other than its normal anatomical location. The prevalence of ectopic thyroid tissue is about 1 per 100,000 to 300,000 persons.^1^ Ectopic thyroid is most common in females, especially in populations of Asian origin.^3^ The thyroid gland originates from the endodermal epithelium of the pharynx. Normally, the thyroid gland appears at about 3 to 4 weeks of gestation and begins to descend in front of the hyoid bone and laryngeal cartilage by the seventh week of gestation. Finally, it reaches a position in front of the trachea.^4^ Pericardial or cardiac ectopic thyroid may develop from abnormal persistent contact between the endodermal thyroid primordium and bulbus cordis.^5^ Ectopic thyroid is very rarely found in the heart. From the first case reported in 1941 to the present, 39 cases have been reported, and the incidence of these cases is predominantly in elderly women. The ectopic thyroid tissue predominantly has been found in the RVOT, followed by the root of the aorta and ascending aorta.^2^

Common symptoms of thyroid cardiac mass are dyspnea and palpitations after activity associated with outflow tract obstruction. Patients would have had ectopic thyroid at birth, but did not develop symptoms until later in life.^6^ The reason for the growth of the mass is not known. One study found that the mass had a blood supply vessel, which was a branch of the left anterior descending artery.^1^ Echocardiography is the primary test for finding the mass, which is further confirmed by CT and magnetic resonance imaging. Ultimately, it is diagnosed by histological analysis after the mass is excised. Intracardial thyroid is classified into 1 of 2 types^7^: type I, the absence of thyroid tissue in its normal anatomical position but in other locations; and type II, the presence of thyroid tissue both in its normal anatomical position and in other locations. Our case was a type II ectopic thyroid with normal thyroxine levels. Surgical resection is the main treatment option,^1^ especially in large masses that cause outflow tract obstruction. Thyroid function should be monitored after surgery, although hyperthyroidism is extremely rare.

## Conclusions

Intracardial thyroid is an extremely rare condition. It occurs preferentially in the RVOT of middle-aged women and may cause outflow tract obstruction. Surgery is safe and effective and there were few postoperative complications in our case.

The ethics committee of the Beijing Anzhen Hospital, Capital Medical University approved the study protocol and publication of data (No.: 2024209X. Date: October 22, 2024). The patient(s) provided informed written consent for the publication of the study data.

## Conflict of Interest Statement

The authors reported no conflicts of interest.

The *Journal* policy requires editors and reviewers to disclose conflicts of interest and to decline handling or reviewing manuscripts for which they may have a conflict of interest. The editors and reviewers of this article have no conflicts of interest.
